# Complete genome sequence of *Subdoligranulum* sp. strain DSM 23681 isolated from the cecum of a broiler chicken

**DOI:** 10.1128/mra.00619-26

**Published:** 2026-09-18

**Authors:** Paul Taillandier, Karine Clément, Tiphaine Le Roy

**Affiliations:** 1 Sorbonne Université, Inserm, Nutrition and Obesities: Systemic Approaches, Nutriomics, Paris, France; 2 Nutrition Department, Assistance-Publique-Hôpitaux de Paris, Pitié-Salpêtrière Hospital, Paris, France; Wellesley College, Wellesley, Massachusetts, USA

**Keywords:** gut microbiome, anaerobes, subdoligranulum, gemmiger, Ruminococcaceae, butyrate, metformin, diabetes, microbiota, chicken

## Abstract

We report the complete genome sequence of strain ic1395 (DSM 23681), an anaerobe previously isolated from the cecum of a broiler chicken and affiliated with the *Subdoligranulum* genus. Its average nucleotide identity with the closest species with standing in nomenclature indicates that this strain represents a divergent and distinct lineage.

## ANNOUNCEMENT

*Subdoligranulum* genus is frequently detected in the human gut microbiota and has repeatedly been associated with a healthy cardiometabolic profile across independent cohorts (1). The abundance of the *Subdoligranulum* genus correlates with metabolic markers (2–4) and has been reported to fluctuate in response to metabolic interventions, particularly metformin treatment (5).

The genus *Subdoligranulum* currently comprises a single species with standing in nomenclature: *Subdoligranulum variabile* (6). Supplementation with *S. variabile* in a murine model of high-fat diet-induced obesity showed no beneficial effect on host metabolism (7). However, *S. variabile* accounts for less than 1% of the total abundance of the *Subdoligranulum* genus in humans, suggesting that the genus-level signal observed in microbiome studies primarily reflects the positive effects of other unresolved members of the lineage (7).

In this context, we obtained *Subdoligranulum* sp. strain ic1395 from the DSMZ collection (DSM23681). This strain was isolated from the cecum of a broiler chicken (*Gallus gallus domesticus*) in Denmark (8). Its 16S rRNA gene sequence is 96.8% similar to that of *S. variabile*, indicating a significant evolutionary divergence within this lineage and highlighting the relevance of whole-genome sequencing to fully resolve the specific functional traits and diversity of this genus.

Strain ic1395 was cultivated for 2 days at 37°C in liquid medium composed of yeast extract (5 g/L), soy peptone (10 g/L), NaCl (1 g/L), K_2_HPO_4_ (1.5 g/L), Na_2_CO_3_ (3 g/L), ornithine (1 g/L), sodium acetate (2 g/L), aspartic acid (1.5 g/L), formic acid (3 mL/L), Tween 20 and 80 (1.5 mL/L), hemin (100 µg/L), fetal bovine serum (30 mL/L), and menadione (2 µg/L). Genomic DNA was extracted by mechanical lysis (bead-beating with 0.1-mm glass beads), followed by phenol-chloroform extraction, ethanol precipitation in the presence of ammonium acetate, RNase treatment, and purification using AMPure XP magnetic beads (Beckman Coulter).

Whole-genome sequencing was then performed; key read quality and assembly metrics are summarized in Table 1.

**TABLE 1:** Summary of PacBio Sequel IIe (HiFi) sequencing output and *de novo* genome assembly metrics for strain ic1395 (DSM 23681)

Category	Metric	Value
Sequencing	System/mode	PacBio Sequel IIe (HiFi)
	Library	SMRTbell
	Facility	Institut Curie sequencing platform (Paris)
	Reads (*n*)	154,851
	Total bases (bp)	1,109,443,303
Read QC	QC assessment	NanoPlot (Galaxy Europe)
	Mean read length (bp)	7,164.6
	Median read length (bp)	6,985
	N50 (bp)	7,736
	Mean Q	30.1
	Median Q	39.2
*De novo* assembly	Assembler	Flye (Galaxy Europe)
	Contigs	1
	Topology	Circular
	Size (bp)	3,143,997
	Estimated coverage	368×
	GC content (%)	58.97%

**Fig 1:**
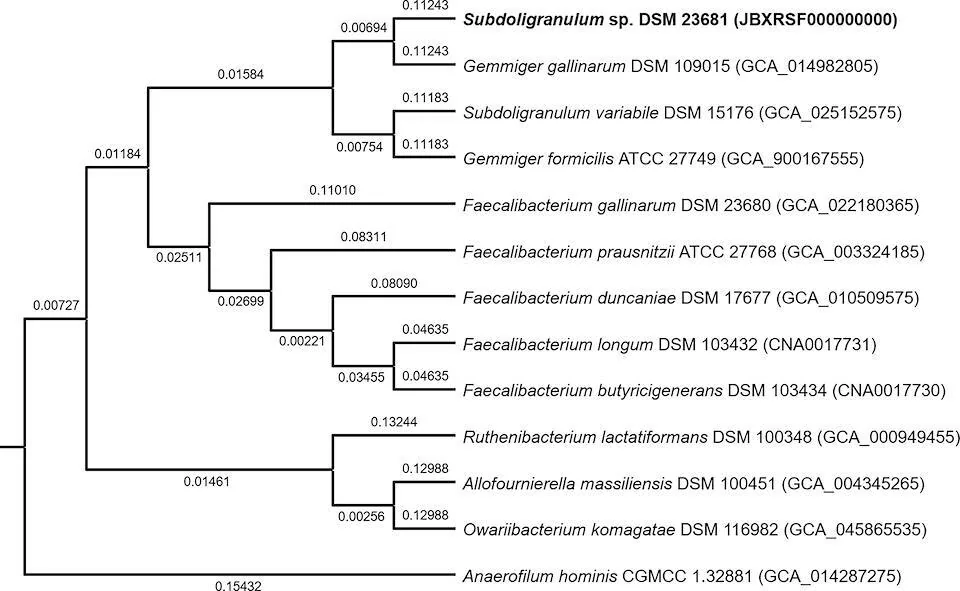
Genome similarity tree based on average nucleotide identity (ANI). Pairwise ANI values were calculated using the OrthoANI algorithm (9) from an all-versus-all genome comparison. ANI values were converted to distances (*D* = 1 − ANI/100), and the tree was inferred using unweighted pair group method with arithmetic mean (UPGMA). Clustering was performed in RStudio (v12.0), and the resulting tree was visualized and graphically refined in MEGA (v12.0.9). Branch lengths are proportional to *D*.

Pairwise ortho-average nucleotide identity (OrthoANI), computed using the OAT_cmd command-line tool implementing the OrthoANI algorithm (9), was performed to determine the phylogenetic relationships between strain ic1395 and neighbor taxa belonging to the *Oscillospiraceae* family (Fig. 1). The highest similarities were observed with *Gemmiger gallinarum* (77.51%), *Subdoligranulum variabile* (77.36%), and *Gemmiger formicilis* (75.21%), whereas all remaining comparisons involving strain ic1395 were <74.4%. Given that these values are below the standard 95%–96% ANI threshold for species delineation, strain ic1395 represents a deeply branching and yet uncharacterized member of the *Gemmiger-Subdoligranulum* clade.

*S. variabile* exhibits genome-wide similarity to *Gemmiger* species within the same order of magnitude as that observed among potential novel *Subdoligranulum* species, highlighting their close phylogenetic relationship. The genus *Gemmiger* was first described in 1976 and comprises two species (10). This genomic proximity is consistent with the Genome Taxonomy Database (R10-RS226), which classifies *Subdoligranulum*-affiliated genomes as *Gemmiger* based on normalized phylogenetic distance (11). Although formal nomenclatural changes remain under the purview of polyphasic taxonomic revisions, our data could contribute to the ongoing discussion about the boundaries of these genera.

## Data Availability

This whole-genome shotgun project has been deposited at DDBJ/ENA/GenBank under accession number JBXRSF000000000. Reads can be accessed in the Sequence Read Archive with accession number SRX33242970.
